# A High-Robust Automatic Reading Algorithm of Pointer Meters Based on Text Detection

**DOI:** 10.3390/s20205946

**Published:** 2020-10-21

**Authors:** Zhu Li, Yisha Zhou, Qinghua Sheng, Kunjian Chen, Jian Huang

**Affiliations:** School of Electronics and Information, Hangzhou Dianzi University, Hangzhou 310000, China; lz1126@hdu.edu.cn (Z.L.); hd_zys@hdu.edu.cn (Y.Z.); chenkunjian@hdu.edu.cn (K.C.); jian.huang@hdu.edu.cn (J.H.)

**Keywords:** pointer meter, deep learning, secondary search, distance method

## Abstract

Automatic reading of pointer meters is of great significance for efficient measurement of industrial meters. However, existing algorithms are defective in the accuracy and robustness to illumination shooting angle when detecting various pointer meters. Hence, a novel algorithm for adaptive detection of different pointer meters was presented. Above all, deep learning was introduced to detect and recognize scale value text in the meter dial. Then, the image was rectified and meter center was determined based on text coordinate. Next, the circular arc scale region was transformed into a linear scale region by polar transform, and the horizontal positions of pointer and scale line were obtained based on secondary search in the expanded graph. Finally, the distance method was used to read the scale region where the pointer is located. Test results showed that the algorithm proposed in this paper has higher accuracy and robustness in detecting different types of meters.

## 1. Introduction

Pointer meters are widely used in petrochemical, electric power and other industries because of their simple structure, convenient use and low cost [1]. Because most of these meters have no digital communication interface, the manual reading method is usually adopted, but the manual detection cost is high and the efficiency is low, which is insufficient to meet the real-time and intelligent monitoring requirements in industry [2]. The automatic reading of pointer meters [3,4,5,6,7] can save a lot of labor and time costs for factories, so it has great practical value [8,9,10].

In the past few years, many researchers have put forward automatic recognition methods of pointer meters based on computer vision technology. Alegria et al. [11] first applied computer vision to meter readings, and developed an automatic calibration system for meter dials, which can automatically read the meter readings. They first used binary and thinning operations, then subtracted the two preprocessed images to extract the pointer, and used Hough transform to fit the pointer line, thus getting and calibrating the readings. Belan et al. [12] proposed a free segmentation method, and realized the calibration of digital and analog measuring instruments. They used radial projection and Bresenham line algorithm to locate the pointer position, thus obtaining the readings of pointer meters and calibrating the meters. Zheng et al. [13] proposed a robust automatic recognition algorithm. MSRCR with color recovery function was used for preprocessing to reduce the influence of brightness, and projection transformation was applied to obtain the front view of the image, and then Hough transform was used to recognize the pointer and get the readings. This algorithm has improved the robustness of meter recognition system to brightness and shooting angle. Gao et al. [14] put forward an adaptive algorithm for verifying automobile dashboard. They first used cascaded HOG/SVM and HOG/MSVM to locate and recognize the digital text of scale value. Contour analysis method was used to extract pointer to eliminate the adhesion between pointer and scale value, and then the region of searching scale line was narrowed according to the spatial relationship between number box and scale line, thus extracting the main scale line. Finally, the angle of each scale line was calculated, and the pointer reading at a certain angle was obtained by Newton linear interpolation to judge whether the dashboard is qualified. Ma et al. [15] proposed a robust recognition method for pointer meter, which can eliminate the influence of surrounding scale line, characters, figures and graphics on the pointer, so as to extract the pointer image more accurately. They first detected the center of rotation of the pointer according to the circularity of the pointer region, then determined the threshold value based on the symmetry of the pointer, so as to accurately divide the image of pointer region. Then, they used RANSAC line drawing method and least square method to recognize the pointer and zero graduation line, respectively, and finally used angle method for reading. Chi et al. [16] put forward an automatic reading method of pointer meter based on computer vision. They first used the method of region growing to locate the dial region and its center. After transformation of coordinates, the scale line was selected according to the frequency of each angle in the angle histogram. Sheng et al. [17] proposed a double Hough space voting algorithm. This algorithm uses scales to fit the meter center, determines the scale region by projection method, and reads the meter according to the distance method. In recent years, many scholars have proposed meter recognition algorithms based on deep learning. Liu et al. [18] used a convolutional neural network to detect the dial, and used the brightness space to detect the specular reflection area. If the area exceeds the set threshold, the inspection robot is controlled to move a distance to prevent a certain light from entering the camera to remove the specular reflection. Then the pointer line is extracted by binary, thinning, and Hough transform, and finally read by the angle method. Zhang et al. [19] used the faster-rcnn to locate and classify the meters in the image, rectified the image according to the slope of the meter edge line, and use the image enhancement algorithm based on Retinex theory to improve the quality of the image. Then obtains the angle of the scale line and the pointer line through the fast refinement algorithm and the straight line fitting algorithm, and finally uses the interpolation method to read. Cai et al. [20] proposed a novel virtual sample generation technology, and then used an end-to-end convolutional neural network to identify the instrument. He et al. [21] first used Mask-RCNN to classify the meter and segmented the pointer image in the meter, and then used the PCA algorithm to fit the pointer line and read through the angle of the pointer.

The methods proposed by Alegria [11], Belan [12] and Gao [14] can achieve a higher accuracy in the verification system with manual control of brightness and shooting angle, but have a poor robustness in industrial fields with more complex shooting environment, such as external substations. In addition, although the methods proposed by Zheng [13], Ma [15], Chi [16] and Sheng [17] have improved the robustness of automatic reading algorithms to a certain extent, these methods are weak in adapting to different meter types, and the proposed algorithms can only be applied to the meter dials with certain characteristics. For example, the extracted vertexes of quadrilateral meter dial were used to make meter rectification in Zheng’s method [13], which is not applicable to those round meters; the method proposed by Ma [15] located the meter center according to the circularity of center of rotation, which is only suitable for those meters with a circular center; the method presented in Chi’s article [16] is also only applicable to meters whose scale line is distributed within the gray scale region. The method proposed by Sheng [17] used scale line to fit the meter center, so the scale image has few characteristics, which is prone to false detection and influence by the image quality.

**Figure 1:**
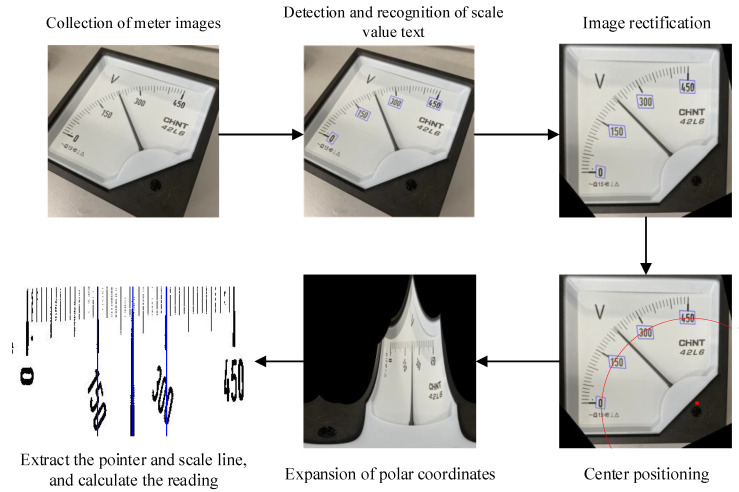
Algorithm flow.

In view of these problems, a highly robust reading recognition method was proposed for pointer meters, which reads meters based on the text region. Because text is the common feature of all meter dials, and text images have richer features than scale line images and can be better located, the method proposed in this paper can realize automatic detection with high precision and robustness. In the algorithm of this paper, the convolutional neural network is used to detect text, because the scale value text in the meter is the same, once the training is completed, the network can be applied to different meters, so when a new meter appears, there is no need to prepare a large amount of training data and retrain the network. Figure 1 shows the flowchart of this algorithm. First, this algorithm applied deep learning to the detection of scale value text. After obtaining an accurate text bounding box, the center coordinates in such box was used for image rectification, thus eliminating the influence of shooting angle on reading recognition. Then, the meter center was determined according to the center point in the text bounding box, so that polar transform can be carried out, and the circular scale line can be converted into a horizontal one. After that, a secondary region search method was proposed to extract the pointer and scale line images of the meter. Finally, the reading between two main scale lines closet to the pointer was obtained by the distance method.

This paper is also superior to the algorithm proposed in the latest paper in some aspects. Liu [18] used the traditional angle method for reading, which requires manual input of the angles of the zero scale line and the full scale line in the image, which is low in efficiency. The algorithm proposed in this paper uses the secondary area search method to directly obtain the main scale line and pointer line. The horizontal coordinate can really be read by algorithm. Zhang [19] used the slope of the edge line of the instrument to correct the instrument image, which is only applicable to instrument panels with straight edges. The algorithm in this paper corrects the image based on the scale value text coordinates. The scale value text is a common feature of different types of instruments, so the algorithm has a wider range of adaptation. Cai [20] used an end-to-end neural network model to directly read the meter. When a new meter appears, a large amount of training data needs to be prepared again and the network needs to be retrained. Algorithm cannot be used flexibly on different instruments. The deep learning introduced in this article is to detect the scale value text which is the common feature of the instrument. Once the training is completed, algorithm can be applied to different instruments. He [21] used Mask-RCNN to obtain the pointer area. For new instruments with different pointer shapes, a large number of data sets need to be prepared and the network needs to be retrained. The algorithm cannot be flexibly applied to different instruments. This article uses the projection of pixel values to locate the horizontal coordinates of the pointer, which is applicable to pointers of different shapes.

**Figure 2:**
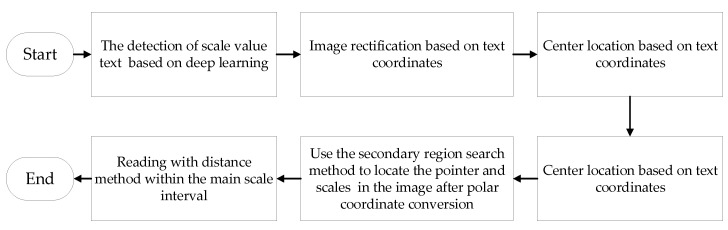
The flowchart of algorithm implementation.

The main contributions of the algorithm proposed in this paper are as follows:

- Deep learning was applied to the detection of scale value text in the meters, which realizes the text coordinate positioning with high precision and robustness, and the text recognition with high accuracy. Also, compared with the distance method of reading from zero scale to full scale, using the recognition result of scale value in the distance method of reading allows a smaller error.
- A novel meter center positioning method was proposed, which locates the meter center according to the position of scale value text. The image of scale value text provides more features than that of scale line, so it can adapt to more complex environments when used to fit the meter center.
- The detection of scale value text was applied to the meter rectification. Since scale value text is a common feature of almost all meters, such design can greatly improve the adaptive ability of the algorithm.
- Based on the position of scale value text, a secondary region search method was proposed to extract the pointer and scale line. This method has effectively solved the problem of pointer shadow, and also eliminated the influence of other objects in the dial on pointer and scale line extraction. The detailed algorithm flowchart is shown in Figure 2.

Although different meter dials should may have different shapes and structures, their scale value texts are usually Arabic numerals. Hence, in this paper, meter rectification, meter center positioning, pointer and scale line extraction are all based on the detection of scale value text, which greatly improves the adaptive ability of the algorithm. The other parts of this paper are arranged as follows: Part 2 introduces the text detection and recognition algorithm based on deep learning, and image rectification based on text bounding box; Part 3 introduces the method of extracting pointer and scale line and meter reading based on secondary region search method; Part 4 illustrates the test results; and the final Part 5 shows the conclusions.

## 2. Image Rectification Based on Text Position

### 2.1. Digital Detection and Recognition of Scale Value

In this paper, the algorithms of meter rectification, meter center positioning, pointer recognition and scale line recognition are all based on the detection of meter scale value, while the traditional meter detection methods are all based on SVM classification [22], which is insufficient to adapt to complex industrial site environment. In order to improve robustness, a neural network was introduced, which makes the text detection and recognition algorithm more robust and adaptable, and also lays a good foundation for the later meter image rectification and reading.

**Figure 3:**
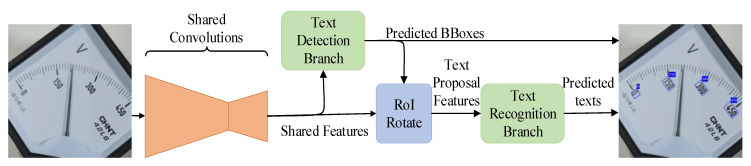
FOTS model of text detection network.

In this paper, a FOTS [23] network was used to detect and recognize the text of scale values in the meter. FOTS is an end-to-end text recognition model, of which the network structure is shown in Figure 3. Firstly, a shared convolution network is used to extract the shared features of the image, and these features are used to determine the position of text region in the detection part. RoIRotate samples this part of features from the shared features according to the position of text region detected in the detection part, which can be used to predicate the text in the recognition part.

**Figure 4:**
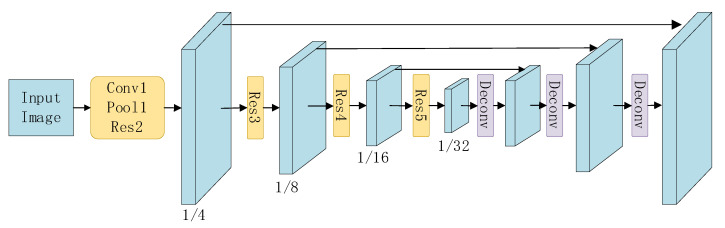
The structure of the shared convolution neural network.

In FOTS, the shared convolution neural network is a U-shaped structure, as shown in Figure 4. It uses ResNet50 [24] for encoding, and then obtains the shared features by decoding through repeated up-sampling, connection operation and two-layer convolution. For points on the feature map, in the detection part, whether these points belong to the text region is firstly predicted, followed by the distance from the points to the four boundaries of the text region and the rotation angle of the text box. After that, a threshold is set to filter the points, and non-maximum suppression is applied to the generated prediction box, finally obtaining multiple region areas. RoIRotate module uses bilinear interpolation sampling to convert the text feature map with indefinite length and certain angle into a feature map without angle but with definite length. In the recognition part, the convolution neural network, which only contracts in height, is firstly used to further encode the feature map inputted, and then the bidirectional LSTM is used to decode the features to generate the final prediction string. The structure of the recognition network is shown in Table 1.

### 2.2. Image Rectification

The image taken by the camera perpendicular to the meter dial is the ideal image. However, in the collection process, it cannot be guaranteed that the camera is always perpendicular to the meter dial, so the image is rectified by projection transformation to reduce subsequent reading errors. Here, we assume that the distance between the meter pointer and the meter plane is much smaller than the distance between the camera imaging surface and the target object. Based on this assumption, we use the same parameters to perform projection transformation on the pointer and meter plane images.

The rules for projection transformation are shown in Equations (1)–(3).

(1) $$(x,y,w^{\prime })=(u,v,w)\times T=(u,v,w)\times \left(\begin{array}{ccc} A_{11} & A_{12} & A_{13}\\ A_{21} & A_{22} & A_{23}\\ A_{31} & A_{32} & A_{33} \end{array}\right)$$

(2) $$\left(X,Y)=(\frac{x}{w^{\prime }},\frac{y}{w^{\prime }}\right)$$

(3) $$\left(U,V)=(\frac{u}{w},\frac{v}{w}\right)$$

wherein, (U,V) is the coordinates of a point in the original image; (X,Y) is the coordinates of the point in the transformed visual plane; (u,v,w) and $\left(x,y,w^{\prime }\right)$ are the expressions of homogeneous coordinate system for (U,V) and (X,Y), respectively; w and $w^{\prime }$ take 1. T is the transformation matrix from the original visual plane to the new visual plane, and it can be uniquely determined according to the coordinates of four pairs of points in these two visual planes. It is easy to obtain the position of scale value text in the ideal image, that is, (X,Y) in Equation (2) is known, and the center coordinates of the text bounding box in the image to be read is taken as (U,V), so as to rectify the meter image.

## 3. Pointer and Scale Extraction

After scale value text positioning and image rectification, the meter is read in the polar coordinate space in this algorithm, which include three steps: (1) Determine the polar coordinates transformation center and expand the polar coordinates; (2) Extract the pointer and scale line; (3) Obtain the reading according to the spatial relation between pointer and scale line. In this part, an adaptive method for determining polar transform center and a method for extracting pointer and scale line based on secondary region search are introduced.

### 3.1. Polar Transform

The pointer meters with higher precision always have a denser scale distribution, which cause greater difficulty in separating the single scale in the curved region, so does the application of angle method in reading. In consequence, it is necessary to carry out polar transform of the meter image, and convert the curved scale into a linear scale whose relative position is easy to calculate. The essence of polar transform is to transform an image from Cartesian coordinate system to polar coordinate system with a certain point in the image as the center, and the correctness of such transformation largely depends on the accuracy of extracting the center. Hence, a center extraction method with great robustness is proposed, i.e., using text bounding box to extract the center.

**Figure 5:**
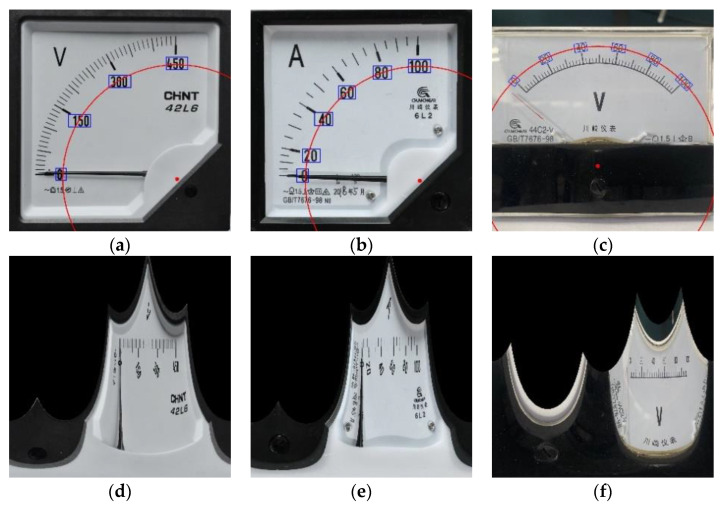
Center fitting and polar coordinate expansion: (**a**–**c**) show the center fitted with the coordinates of scale values; (**d**–**f**) show the polar coordinate expansion of meter images.

The scale values of the meter are distributed on an arc, of which the center is the rotation center of the meter pointer. Therefore, the center of polar transform can be determined by fitting the arc with the coordinates of scale value texts as the data points. After the accurate coordinates of the scale value text box are obtained, the center coordinates are obtained by least square fitting method [25]. After that, the instrument image is converted into a polar coordinate system according to the Equations (4) and (5).

(4) $$\rho =\sqrt{{\left(x_{o}-C_{x}\right)}^{2}+{\left(y_{o}-C_{y}\right)}^{2}}$$

(5) $$\theta =\arctan(\frac{y_{o}-C_{y}}{x_{o}-C_{x}})$$

wherein, $x_{o}$, $y_{o}$ is the abscissa and ordinate in the original coordinate system; ρ and θ are the polar radius and polar angle in the polar coordinate system; $C_{x}$ and $C_{y}$ are the poles in the polar coordinate system. After the polar radius and polar angle of each pixel in the polar coordinate system are worked out, the polar radius and polar angle are taken as the abscissa and ordinate and expanded in the rectangular coordinate system. Figure 5 shows the process of obtaining the center and expanding the polar coordinates based on the coordinates of scale value texts. 

### 3.2. Pointer and Scale Extraction

**Figure 6:**
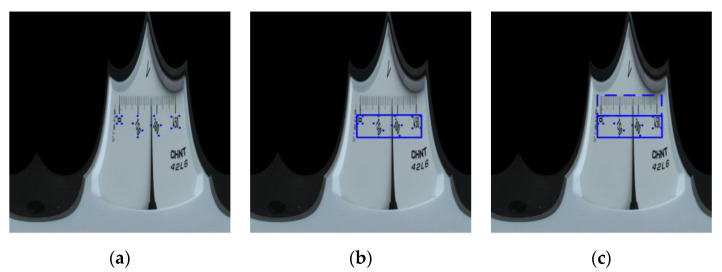
Determination of primary search region: (**a**) Point set composed of vertices of the scale value text positing box; (**b**) Scale value text region; (**c**) Primary search region obtained by expanding the scale value text region.

After polar transform, the coordinates of point $\left(x_{o},y_{o}\right)$ in the original coordinate system is changed to $\left(\frac{W}{360}\times \theta ,H-\rho \right)$, where W and H are the width and height of the image after polar transform, and θ and ρ represent the polar angle and polar radius, respectively, which can be obtained by Equations (4) and (5). By calculating the coordinates of all vertices of the scale value text box in the original image after polar transform, the point set $A\{P_{1},P_{2},\ldots ,P_{m}\}$ of all vertices of the scale value text box in the new image can be obtained, as shown in Figure 6a. From Equations (6)–(9), the region R1 of whole scale value texts is obtained, where $X_{R1}$, $Y_{R1}$, $W_{R1}$, $H_{R1}$ represent the abscissa and ordinate of vertex in the left upper corner of this region, and its width and height, respectively; $x_{1},x_{2},\ldots ,x_{m}$ is the abscissa of points in the point set A, and $y_{1},y_{2},\ldots ,y_{m}$ is the ordinate of the same. Figure 6b shows the R1 region.

(6) $$X_{R1}=\min(x_{1},x_{2},\ldots ,x_{m})$$

(7) $$Y_{R1}=\min(y_{1},y_{2},\ldots ,y_{m})$$

(8) $$W_{R1}=\max(x_{1},x_{2},\ldots ,x_{m})-\min(x_{1},x_{2},\ldots ,x_{m})+1$$

(9) $$H_{R1}=\max(y_{1},y_{2},\ldots ,y_{m})-\min(y_{1},y_{2},\ldots ,y_{m})+1$$

R1 region is expanded so that $h^{\prime }=2h$ is true. Wherein, h is the height of R1; h′ is the height of R1 after expansion. The region after expansion is the primary search region ROI_1_, as shown in Figure 6c.

**Figure 7:**
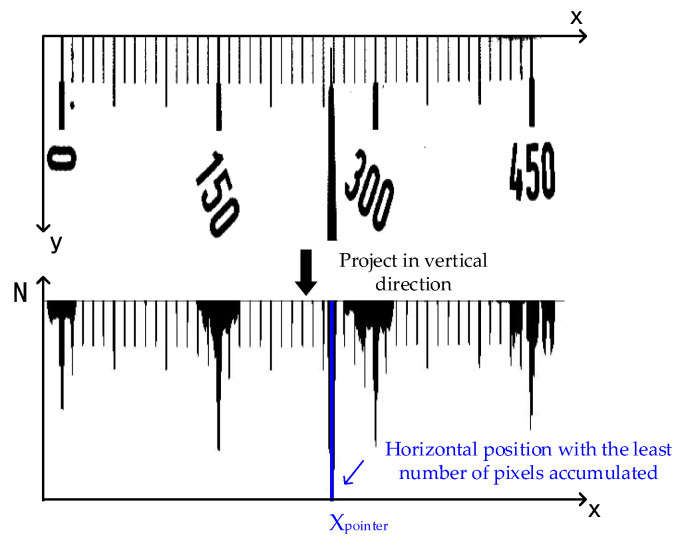
Pixel projection.

In the primary search region, the image is firstly subject to threshold segmentation by the Otsu method and transformed into binary images, and then the white pixels in each column of the image are projected on the X-axis, and the horizontal position with the least accumulated number of pixels is found, as shown in Figure 7. The lower image is the one after projection, and $X_{\mathrm{pointer}}$ is the horizontal position with the least number of pixels, that is, the horizontal position where the pointer is located. Although the pointer shapes vary with the meter types, it is always the same that the number of pixels projected must be the least at the horizontal position of the pointer. Consequently, the horizontal coordinate of the pointer obtained by projection has high accuracy and robustness.

**Figure 8:**
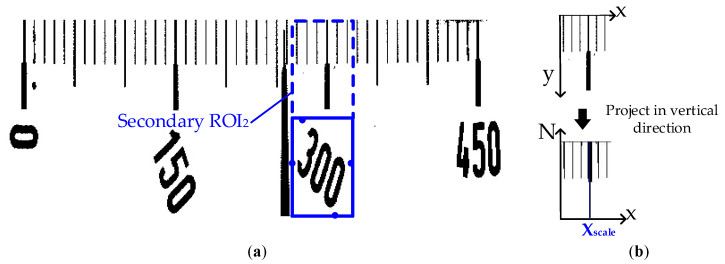
Scale line of secondary region search: (**a**) Secondary search region; (**b**) Region projection.

The next step is the extraction of scale line. Compared with the pointer, the scale line has less obvious features and is more prone to the influence of other objects in the meter dial, so the search range is further narrowed on the basis of primary search region to extract the scale line. According to the position of scale value texts, the secondary search region can be obtained in the following steps: The region containing the scale value text bounding box can be obtained based on the vertexes of such text box, and then a same region as this one is formed on its above, as shown in Figure 8a, and the secondary search region ROI_2_ is therefore obtained. Another vertical projection is conducted in the secondary search region. The white pixels in each line of the image are projected onto the X-axis, and the horizontal position with the least accumulated number of pixels is found, as shown in Figure 8b. $X_{\mathrm{scale}}$ is the horizontal position with the least number of pixels, i.e., the horizontal position of the main scale line.

**Figure 9:**
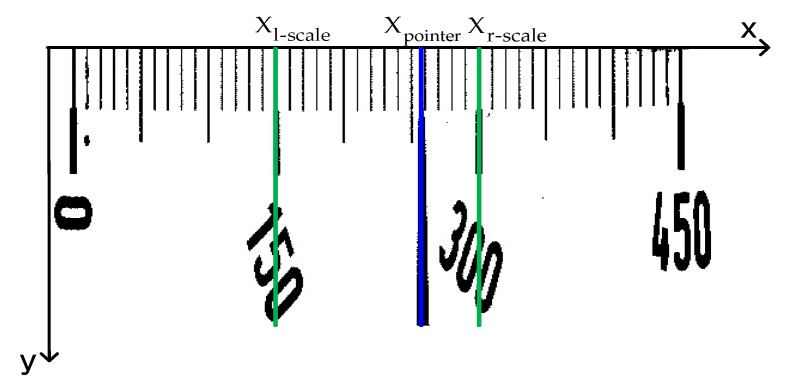
The horizontal coordinates of the pointer and main tick mark in the primary search region.

The readings are calculated in the primary search region. The distance method in the horizontal direction is used for reading. In the pixel coordinate system of the primary search region, the horizontal coordinate $X_{\mathrm{pointer}}$ of the pointer and the horizontal coordinates $X_{\text{l-scale}}$ and $X_{\text{r-scale}}$ of the scale lines corresponding to the scale value text on both sides of the pointer are worked out, as shown in Figure 9. And reading is performed according to Equation (10).

(10) $$V=V_{l}+\frac{X_{\mathrm{pointer}}-X_{\text{l-scale}}}{X_{\text{r-scale}}-X_{\text{l-scale}}}\times (V_{r}-V_{l})$$

wherein, V is the final reading; $V_{r}$ is the scale value corresponding to the scale line on the right side of the pointer, and $V_{l}$ is the scale value corresponding to the left side.

## 4. Experiments

**Figure 10:**
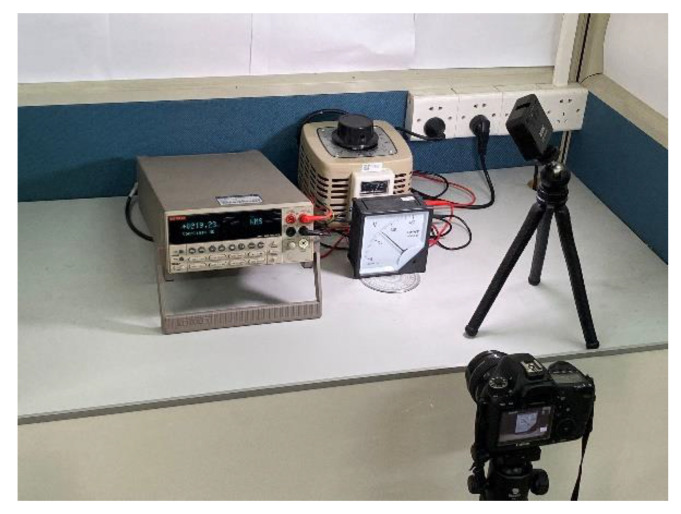
Photograph of the testbed.

**Figure 11:**
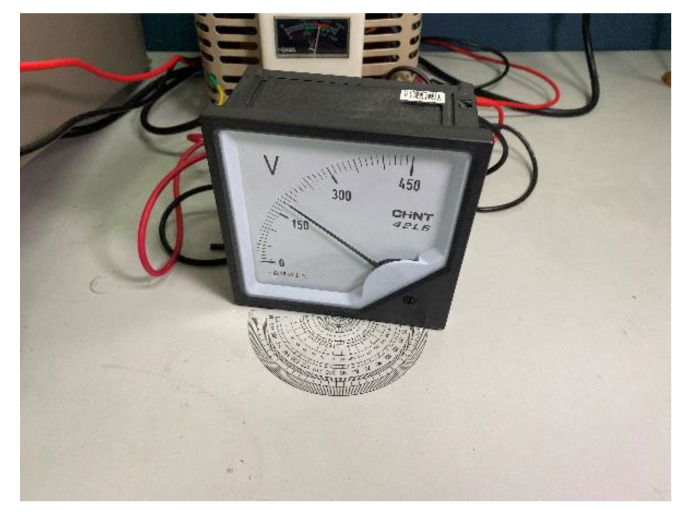
Setting of shooting angle.

The proposed algorithm is evaluated and compared with previous algorithms in this part. The results of scale value text detection, image rectification and extraction of pointer and scale are described in turn. The proposed algorithm is established by using tensorflow platform and opencv library, and is tested on a host with 3.6 GHz Intel Corei 7 processor and 32 GB memory. A test platform with real instruments is established, which includes power supply, meters, multimeter, light source and camera, as shown in Figure 10. Based on the platform, this article establishes two data sets, one of which is used to evaluate the performance of the algorithm, and to compare the algorithm in this article with other algorithms. The images in this dataset include three meter types. And it combines instrument images under different conditions (such as uniform lighting, strong light exposure, shadows and different shooting angles). Each meter has 50 images in each case. Table 2 shows this data set. The other data set is mainly to verify the effect of shooting angle on readings. The data set contains images taken when the angles of each meter are −60, −45, −30, −15, 0, 15, 30, 45 and 60, as shown in Figure 11. All the instrument images in the data set recorded the real values measured by the multimeter when they were collected. The data set for this article is currently not public.

### 4.1. Scale Value Text Detection and Image Rectification

The public dataset SynthText [26] is used to pre-train the end-to-end text detection and recognition network, and then the tagged meter dataset is used to fine-tune the model. The training method is gradient descent in small batch, and data size of each batch is 64, with interaction of 100 cycles. The initial learning rate is 0.001, and is in an exponential attenuation with the attenuation rate of 0.95. Data enhancement technique is adopted in the training, including cropping, rotating, hue changing and Gaussian noise.

**Figure 12:**
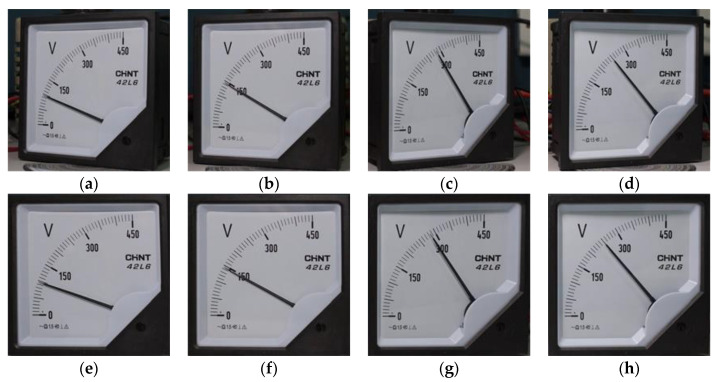
Test results of image rectification algorithm: (**a**–**d**) are the meter image under different shooting angle; (**e**–**h**) are the results after rectification.

To verify the feasibility and robustness of the image rectification algorithm, this algorithm is applied to meter images under different shooting angles. Figure 12e–h shows the transformation results of Figure 12a–d. The test results demonstrated that as long as the meter has the features of scale value text on its dial, the parameters of projection transformation matrix can be obtained based on the text, and the image after rectification can be obtained.

### 4.2. Extraction of Pointer and Scale Line

**Figure 13:**
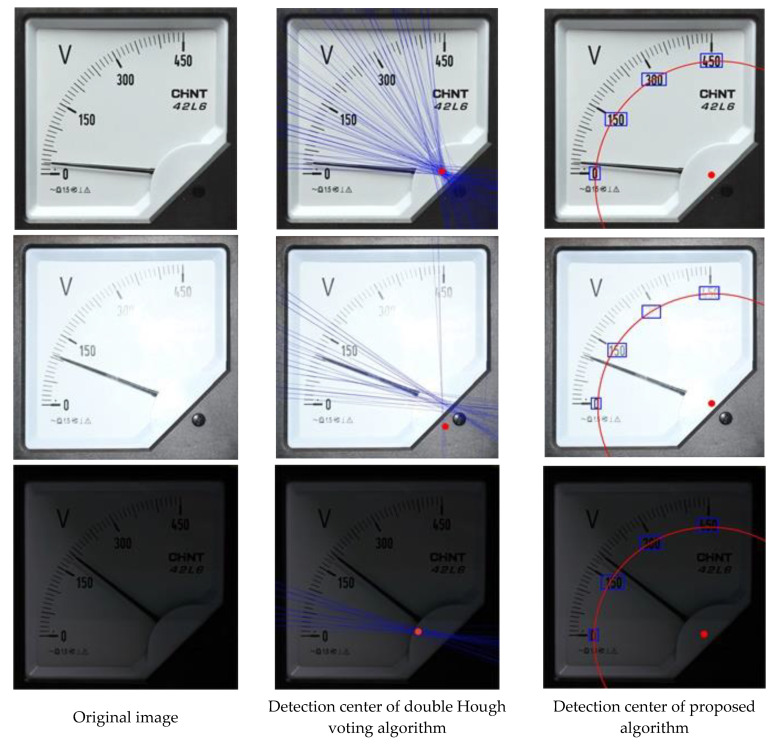
Detection results of meter image center

This section first describes the results of image polar transform. The accuracy of polar transform directly affects the accuracy of extracting pointer and scale line, and the extraction of meter center is the key of polar transform. A circularity-based center extraction method was proposed by Ma [15], but this method is only applicable to certain centers with circular features. A center determination method based on double Hough transform was presented by Sheng [17]. This algorithm is tested in the test set in this paper, and is compared with proposed algorithm method based on scale value text coordinates. Table 3 shows the test results of recognizing the meter center with double Hough transform voting algorithm and proposed algorithm, respectively. Manual measured values and Recognition values represent the center coordinates, and error represents the distance between the recognition values and manual measured values. The pixels of the image are 2000 × 2000. From the table, the recognition results of the 6th, 7th and 9th meter images with double Hough transform voting algorithm have a larger error, and it is analyzed and found that this algorithm cannot accurately fit the center under strong light and shadow. This is because the features of scale line in the meter image are not obvious under strong light and shadow, so that the center cannot be fitted with enough fitting points. In this paper, text is used to fit the center instead of scale line. Since the feature quantity of text is far greater than that of scale line, the center can be accurately located either under strong light or shadow. Figure 13 shows such differences.

**Figure 14:**
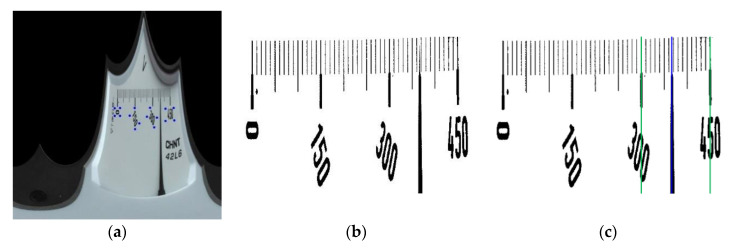
Positioning of pointer and scale line: (**a**) Point set of vertexes of scale value text coordinates in the meter image after polar transform; (**b**) Primary search region; (**c**) Horizontal coordinates of the pointer and main scale lines on both sides in the primary search region.

Polar transform is performed after the center is obtained. The curved scale region is transformed into a linear one, and then the pointer and scale are extracted from the image after transformation. Instead of using the method of fitting scale line and pointer line to extract them, projection is carried out in the two narrowed ROI regions, and the horizontal position of pointer and scale line is determined to get the reading, as shown in Figure 14.

The result of automatic reading is compared with the real value to verify the accuracy of meter detection algorithm. Take the reading measured by the multimeter as the real value. The result of automatic reading algorithm is taken as the test value. The reference error is calculated according to the Equation (11).

(11) $$\gamma_{m}=\frac{x-x_{0}}{x_{m}}\times 100\%$$

wherein, x is the interpretation value of the algorithm; $x_{0}$ is the real value; $x_{m}$ is the meter full-scale value. Proposed algorithm and other algorithms [14,15] are used for the reading of different meters. Table 4 shows part of the reading results of the meter image reading using the algorithm proposed in this paper and other algorithms. It can be seen that the algorithm proposed in this article has higher accuracy than the other two algorithms, and it can also be seen that the algorithm proposed in this paper is more robust to the environment and shooting angle. The reading error of the different shooting angles image is larger than that of other environmental reading errors. The reason is analyzed because even if the image is rectified, the position of the pointer is still different from that in the front view. However, this article has realized the rectification of the instrument, and its accuracy has been greatly improved compared with other reading algorithms. Table 5 shows the average relative error of the proposed algorithm and the other two algorithms [14,15] for three different types of meter readings. It can be seen that the algorithm proposed in this paper can achieve better accuracy for different meters and has good adaptive capabilities.

### 4.3. Analysis of the Error

**Figure 15:**
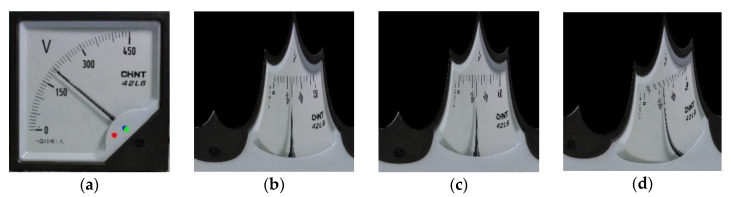

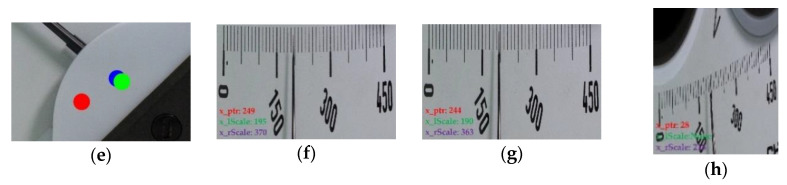
The result of polar coordinate transformation with different coordinates as the center: (**e**) is an enlarged view of (**a**), among them, the blue point is the accurate meter rotation center; the green point is the center with 15 pixel distance error; the red point is the center with a distance error of 95 pixels; (**b**) The result of polar coordinate transformation centered on the correct rotation center; (**c**) The result of polar coordinate transformation centered on the center with 15 pixel distance error; (**d**) The result of polar coordinate transformation centered on the center with 95 pixel distance error; (**f**–**h**) The primary search region ROI_1_ obtained from Figure 15b–d.

This section analyzes the error caused by the circle center fitting, and the result is shown in Figure 15. Figure 15a is the accurate rotation center and the center coordinates obtained by the algorithm; the blue point is the accurate rotation center obtained by the intersection of pointers with different rotation angles. The pixel distance between the green coordinate and the blue coordinate is 15 pixels, and the pixel distance between the red coordinate and the blue coordinate is 95 pixels. Figure 15b is the result of polar coordinate transformation centered on the correct rotation center; Figure 15c is the result of polar coordinate transformation with the center of the 15-pixel distance error as the center; Figure 15d is the result of polar coordinate transformation with the center of the 95-pixel distance error as the center; Figure 15f–h show the primary search region ROI_1_ obtained from Figure 15b–d. The figure also shows the horizontal coordinates of the pointer and the main tick mark obtained by secondary search algorithm. The readings can be calculated according to Equation (10) to be 196.29 V, 196.82 V, and 0 V respectively. It can be seen from the results that the 15-pixel circle center fitting error will not have a large impact on the image after the polar coordinate transformation, nor will it introduce a large reading error.

**Figure 16:**
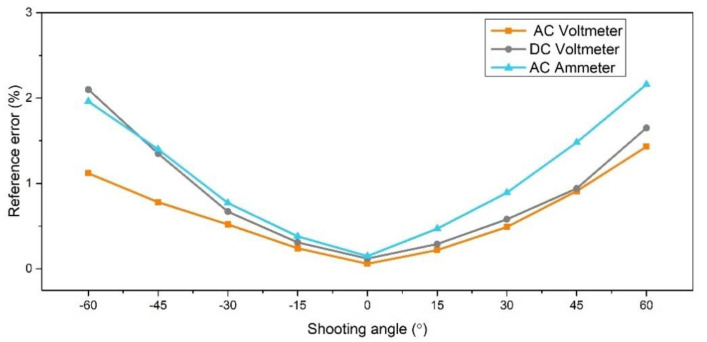
The effect of shooting angle on reading error

We tested the images in the second data set and calculated the error. The relationship between shooting angle and error is shown in Figure 16. It can be seen from the chart that the greater the shooting angle, the greater the reading error caused, which is caused by the fact that the pointer and the meter are not on the same plane. When the shooting angle is within 30 degrees, the reading error is within 1%, and when the angle is greater than 30 degrees, the error is greater than 1%.

## 5. Conclusions

According to the spatial distribution pattern of scale value text and scale region of pointer meters, an automatic reading algorithm of pointer meters based on text detection is proposed, which has high robustness and adaptability. First of all, deep learning is applied to detect and recognize scale value text in the meter dial. Then, the image is rectified and meter center is determined based on the coordinates of scale value text. Next, the curved scale region is transformed into a linear scale region by polar transform, and secondary region search is realized based on the position of scale text, thus obtaining the horizontal positions of pointer and scale line. Finally, the distance method was used to read the scale region where the pointer is located. Just input an ideal image of this type of meter for the algorithm to perform image rectification, and the algorithm can realize automatic detection.

Proposed algorithm has been verified on images of different meter dials. The results demonstrate that this algorithm is robust and highly adaptative to the light and shooting angle. As long as the meter dial has features of scale value text, this algorithm can be used for detection. Also, the accuracy of this algorithm is proven by comparing with state-of-the-art. In the future study, the focus will be on realizing an end-to-end meter detection algorithm. In addition, consider the application of GAN network to image quality improvement. Efforts will also be made to resolve reading errors caused by the fact that the pointer and the dashboard are not on the same plane when the shooting angle is large.

## Figures and Tables

**Table 1:** The structure of the text recognition branch.

Type	Kernel [Size,Stride]	Out Channels
conv_bn_relu	[3,1]	64
conv_bn_relu	[3,1]	64
height-max-pool	[(2,1),(2,1)]	64
conv_bn_relu	[3,1]	128
conv_bn_relu	[3,1]	128
height-max-pool	[(2,1),(2,1)]	128
conv_bn_relu	[3,1]	256
conv_bn_relu	[3,1]	256
height-max-pool	[(2,1),(2,1)]	256
bi-directional_lstm		256
Fully-connected		|S|

**Table 2:** The introduction to the data set used in this article.

Types of Pointer Meter	Range	Image Size	Number of Images
AC Voltmeter	450 V	2000 × 2000	200
AC Ammeter	100 A	2000 × 2000	200
DC Voltmeter	100 V	2000 × 2000	200

**Table 3:** The result of meter center recognition using double Hough space voting and proposed algorithm.

Image Number	Manual Measured Values	Double Hough Space Voting		Proposed Model	
		Recognition Values	Error (Pixel)	Recognition Values	Error (Pixel)
1	[1465,1463]	[1480,1395]	69.63	[1473,1469]	10.00
2	[1470,1466]	[1485,1455]	18.60	[1480,1475]	13.45
3	[1476,1480]	[1454,1437]	48.30	[1486,1472]	12.81
4	[1469,1462]	[1416,1449]	54.57	[1475,1465]	6.71
5	[1469,1463]	[1470,1439]	24.02	[1460,1464]	9.06
6	[1473,1469]	[1271,1484]	202.56	[1462,1501]	33.84
7	[1478,1477]	[1526,1715]	242.79	[1493,1496]	24.21
8	[1478,1480]	[1493,1520]	42.72	[1502,1497]	29.41
9	[1474,1476]	[1523,1311]	172.12	[1476,1477]	2.24
10	[1473,1469]	[1422,1546]	92.36	[1464,1469]	9.00
Average			96.767		15.07

**Table 4:** A part of test results of different methods for reading recognition.

Shooting Environment	Number	Reading by Multimeter (V)	Ref. [14] (V)	Reference Error (%)	Ref. [15] (V)	Reference Error (%)	Proposed Algorithm (V)	Reference Error (%)
uniform illumination	1	23.27	25.39	0.47	25.70	0.54	24.31	0.23
	2	148.91	147.29	0.36	146.53	0.53	150.31	0.31
	3	204.60	202.71	0.42	201.81	0.62	203.07	0.34
	4	367.56	369.23	0.37	369.86	0.51	368.87	0.29
strong light exposure	5	58.10	61.61	0.78	62.11	0.89	56.21	0.42
	6	106.89	111.08	0.93	111.03	0.92	108.60	0.38
	7	219.23	215.36	0.86	215.50	0.83	217.25	0.44
	8	274.26	270.98	0.73	270.75	0.78	272.69	0.35
shadowing	9	124.53	127.91	0.75	128.67	0.92	125.84	0.29
	10	188.72	192.68	0.88	192.50	0.84	190.34	0.36
	11	248.23	251.29	0.68	251.65	0.76	249.67	0.32
	12	302.38	300.11	0.50	298.56	0.85	301.17	0.27
different shooting angles	13	100.03	92.56	1.66	90.87	2.04	95.13	1.09
	14	148.79	143.34	1.21	142.46	1.41	145.01	0.84
	15	247.05	251.96	1.09	253.42	1.42	249.89	0.63
	16	281.26	286.33	1.13	287.32	1.35	284.76	0.78

**Table 5:** The reading error of the proposed algorithm and the other two algorithms on different types of meters.

Shooting Environment	Types of Pointer Meter	Average Relative Error (%)		
		Proposed Algorithm	Ref. [14]	Ref. [15]
uniform illumination	AC Voltmeter	0.295	0.402	0.522
	AC Ammeter	0.343	0.496	0.613
	DC Voltmeter	0.369	0.517	0.596
strong light exposure	AC Voltmeter	0.387	0.769	0.846
	AC Ammeter	0.419	0.845	0.825
	DC Voltmeter	0.401	0.863	0.872
shadowing	AC Voltmeter	0.346	0.755	0.845
	AC Ammeter	0.423	0.799	0.813
	DC Voltmeter	0.376	0.723	0.864
different shooting angles	AC Voltmeter	0.832	1.324	1.621
	AC Ammeter	0.953	1.467	1.694
	DC Voltmeter	0.866	1.332	1.637

## Acronyms

The following acronyms are used in this manuscript:

MSRCR	Multi-Scale Retinex with Color Restoration
HOG	Histogram of Oriented Gradient
SVM	Support Vector Machine
MSVM	Multiple Support Vector Machine
RANSAC	Random sample consensus
LSTM	Long Short-Term Memory
AC	Alternating Currents
DC	Direct Current
ROI	Region of Interest
GAN	Generative Adversarial Networks
